# Heat-induced morphological changes in silver nanowires deposited on a patterned silicon substrate

**DOI:** 10.3762/bjnano.15.39

**Published:** 2024-04-22

**Authors:** Elyad Damerchi, Sven Oras, Edgars Butanovs, Allar Liivlaid, Mikk Antsov, Boris Polyakov, Annamarija Trausa, Veronika Zadin, Andreas Kyritsakis, Loïc Vidal, Karine Mougin, Siim Pikker, Sergei Vlassov

**Affiliations:** 1 Institute of Technology, University of Tartu, Nooruse 1, 50411 Tartu, Estonia; 2 Institute of Solid State Physics, University of Latvia, Kengaraga 8, LV-1063 Riga, Latvia; 3 Estonian Military Academy, Riia 12, 51010 Tartu, Estonia; 4 Institute of Materials Science of Mulhouse, CNRS – UMR 7361, University of Haute-Alsace, France; 5 Institute of Physics, University of Tartu, W. Ostwaldi 1, 50411 Tartu, Estonia

**Keywords:** diffusion, finite element method, heat treatment, molecular dynamics simulations, morphological changes, scanning electron microscopy, silver nanowires

## Abstract

Metallic nanowires (NWs) are sensitive to heat treatment and can split into shorter fragments within minutes at temperatures far below the melting point. This process can hinder the functioning of NW-based devices that are subject to relatively mild temperatures. Commonly, heat-induced fragmentation of NWs is attributed to the interplay between heat-enhanced diffusion and Rayleigh instability. In this work, we demonstrated that contact with the substrate plays an important role in the fragmentation process and can strongly affect the outcome of the heat treatment. We deposited silver NWs onto specially patterned silicon wafers so that some NWs were partially suspended over the holes in the substrate. Then, we performed a series of heat-treatment experiments and found that adhered and suspended parts of NWs behave differently under the heat treatment. Moreover, depending on the heat-treatment process, fragmentation in either adhered or suspended parts can dominate. Experiments were supported by finite element method and molecular dynamics simulations.

## Introduction

Metal nanowires (NWs) are promising key elements in a wide range of applications, including solar cells [1], smart windows [2], flexible sensors [3], touch screens [4], biocompatible polymer binders [5], temperature sensing [6], medical materials [7], and key elements of nanoscale devices [8]. When deposited on a transparent substrate in the form of a low-density mesh, metal NWs can provide electrical conductivity while retaining sufficient transparency. The growing demand for transparent conductive materials has stimulated numerous studies aimed at the design, preparation, and characterization of such materials [9–10]. Silver NWs are among the most extensively studied materials for NW-based transparent electrodes. High-quality Ag NWs can be synthesized relatively easily in large quantities, with precise control over their length and diameter [11–12]. Compared to indium tin oxide (ITO), which currently serves as the industry standard for transparent conductive films, the Ag NW network is significantly more mechanically flexible and offers a broader optical transmittance range that extends well beyond the visible region [13–14]. Another related application of Ag NW networks is in highly flexible transparent film heaters [15]. In recent years, Ag NWs have garnered attention as a key element in neuromorphic computing devices [16].

In the context of the applications mentioned, Ag NWs are subjected to elevated temperatures caused by Joule heating [17]. Moreover, after depositing NWs onto a substrate, heat treatment at temperatures around a few hundred degrees Celsius is often employed to eliminate the surfactant used during synthesis [18–19]. The melting temperature of silver is 962 °C, which is significantly higher than the temperatures required to remove organics. However, when the size of the structures is reduced to the nanoscale, metals exhibit distinct behavior at elevated temperatures compared to their larger counterparts [20–21]. Generally, a reduction in the melting point occurs as the size and dimensionality of the nanostructures decrease [20,22–23]. This phenomenon is closely related to the variation of surface energy with size [24]. For instance, the melting temperature can decrease by several hundred degrees for structures smaller than 10 nm [25]. In practical applications, the diameters of Ag NWs are typically significantly larger. However, during prolonged heat treatment (lasting minutes or more), surface atom diffusion can lead to morphological changes in NWs even at temperatures several hundred degrees below the melting point of the material [26–27]. Sintering of Ag and Au NWs at intersections can occur at temperatures as low as 125–200 °C within minutes [27–28]. This effect enhances the electrical conductivity of the NW network by improving electrical contacts between individual NWs [28–30]. However, further temperature increases may cause NWs to split into shorter fragments – a process often attributed to Rayleigh instability and energy minimization via spheroidization [28,31]. It has been demonstrated that various coatings can effectively protect metallic NWs by suppressing surface diffusion [32–34].

The kinetics of diffusive processes in NWs are tightly related to the surface energies of the system. Both Ag and Au NWs have a pentagonal cross-section, meaning that for NWs deposited on a flat substrate, 1/5 of the NW surface is in contact with the substrate [35]. This aspect should unavoidably have an influence on the total surface energy of NW. Therefore, in addition to parameters such as temperature, time, and geometry of NWs, contact with the substrate can potentially have a considerable effect on heat-induced changes in NWs. Understanding the fragmentation behavior of metal NWs under different conditions and on various substrates could improve the degree of control in cost-effective production methods for various novel applications where arrays of metal nanostructures are used, such as surface-enhanced Raman spectroscopy substrates [36–38].

In this work, we deposited Ag NWs on specially patterned silicon (Si) substrates, so large fractions of NWs are partially suspended over the holes. Samples were then heated to different temperatures in air, and the behavior of suspended as opposed to the adhered part under heating was compared. Experiments are supplemented with molecular dynamics (MD) and finite element method (FEM) simulations.

## Materials and Methods

### Preparation of samples

Silver NWs with a nominal diameter of 120 nm and length of tens of micrometers were purchased from Blue Nano, Inc. These NWs have a pentagonal cross-section and a five-fold twinned inner structure. More details on the structure and properties of these NWs can be found in our previous works [35,39].

The patterned silicon substrates with square holes were prepared from (100) silicon wafers (Semiconductor Wafer, Inc.) with 50 nm thermal oxide in four steps as follows: 1) conventional optical lithography process to produce the desired pattern in a photoresist on the wafer; 2) selective removal of SiO_2_ using buffered HF solution in order to replicate the resist pattern in the oxide layer; 3) silicon etching in tetramethylammonium hydroxide (TMAH) solution at 90 °C to create the etch pits; 4) rinse in HF to remove the remaining SiO_2_. The resulting substrates had rectangular holes with a side length in the order of a few µm (3.6 to 5.3 µm) and a depth in the order of several hundreds of nanometers. The distance between holes, depending on the direction, varied from hundreds of nanometers to several micrometers. The slope of the sidewalls of the holes relative to the main surface of the silicon is 54.7 degrees, which corresponds to the angle between (111) and (001) planes in Si.

**Figure 1:**
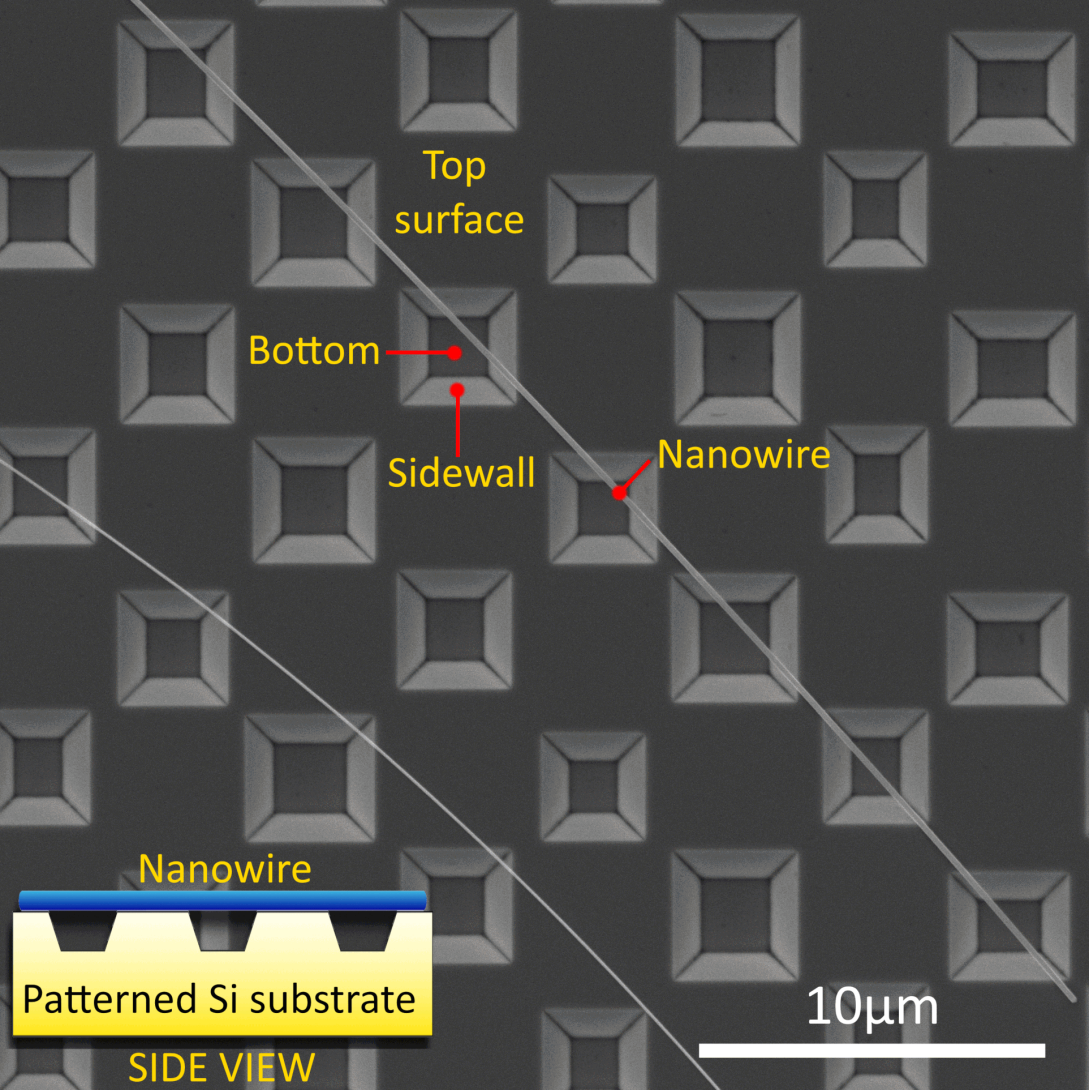
Single Ag NW suspended over several holes etched in a Si substrate.

Samples for heat-treatment studies were prepared by drop-casting Ag NWs onto the patterned substrates from a solution. Since the width and period of the holes were intentionally made much smaller than the average length of NWs, many NWs simultaneously crossed several holes. Such configuration is highly beneficial as it enables to study the effect of the substrate on the same NW by comparing the behavior of suspended as opposed to adhered parts (Figure 1).

### Heat treatment and characterization

Heat-treatment experiments were carried out in a muffle furnace (NABERTHERM, L-091H1RN-240). Samples were placed into the furnace that was already preheated to the target temperature, then they were removed after 10 min from the hot furnace, and were naturally cooled in air at room temperature. The heating time (10 min) was chosen for comparison with the work by Vigonski et al. [27] who used the same Ag NWs and heating methods for heat-treatment experiments of a flat Si substrate.

**Figure 2:**
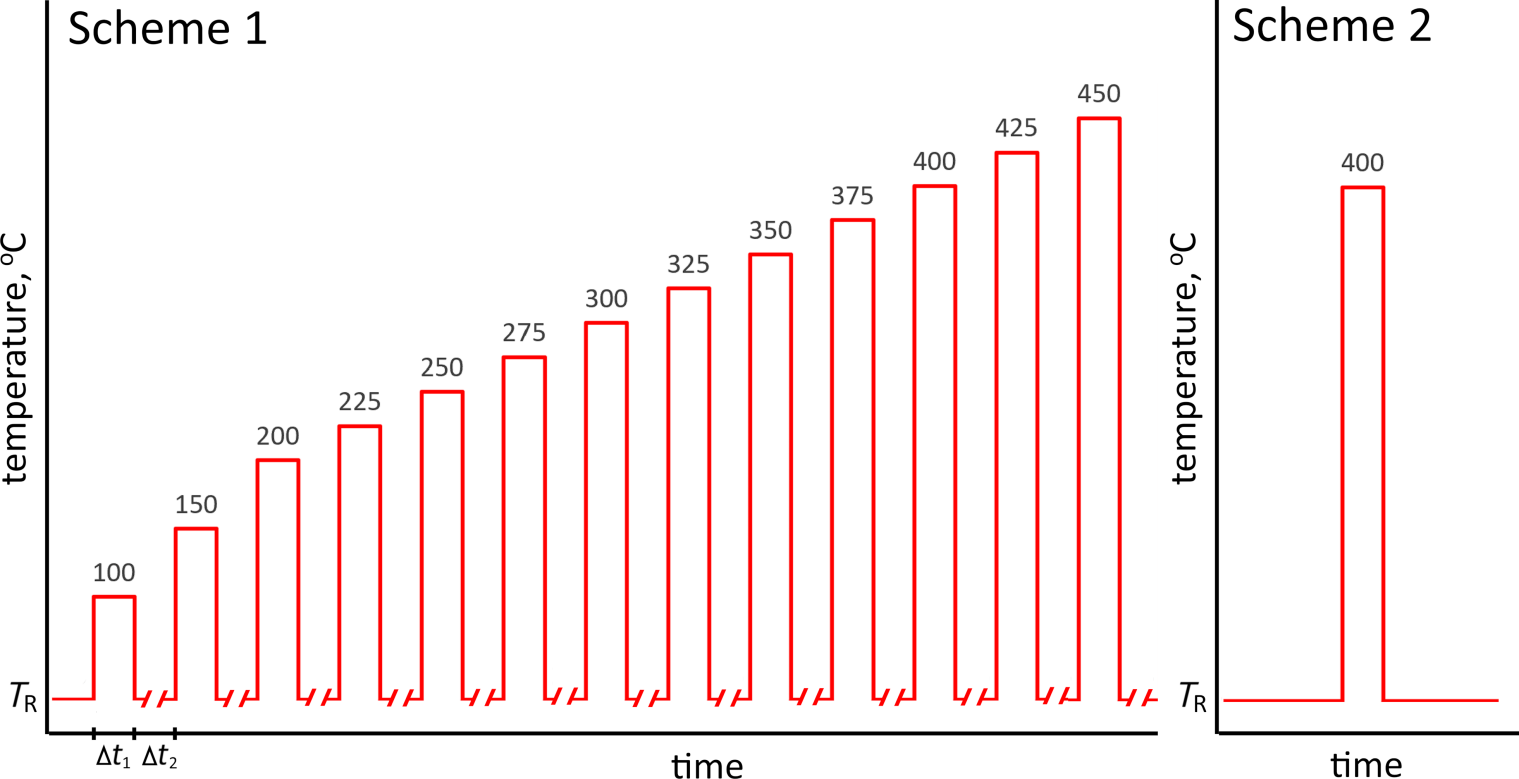
Heating schemes used for heat treatment of Ag NWs. *T*_R_ stands for room temperature. Δ*t*_1_ = 10 min is a heat treatment time, Δ*t*_2_ ≈ 1 h is the rest time.

Two heating schemes were implemented: In the scheme 1 (Figure 2), heating was applied in 10 min cycles at fixed temperatures followed by cooling to room temperature. The temperature of the first cycle was 100 °C and in each following step, it was increased by 50 °C until 200 °C, and then by 25 °C increments until reaching 450 °C. In the scheme 2 (Figure 2), freshly made samples were heated in a single step for 10 min at a target temperature chosen based on the results from the scheme 1.

Approximately one hour waiting time between cycles was chosen to give enough time for taking series of scanning electron microscopy (SEM, FEI, Nanosem 450) images after each heating cycle.

Additionally, a separate series of transmission electron microscopy (TEM) experiments were performed to study the effect of heat treatment on the inner structure of NWs. Two transmission electron microscopes (Tecnai GF20, FEI and JEOL microscope, model ARM-200F) were used at a 200 kV acceleration voltage.

### Simulations

The extent and distribution of mechanical stresses induced by thermal expansion of Ag NWs and a substrate during heat treatment from room temperature to 673.15 K were simulated by FEM in Comsol Multiphysics 5.6. The structural configuration involved a pentagonal Ag NW positioned above a rectangular hole on an Si substrate. The NW was securely affixed to the substrate, while the overhanging segment retained freedom of movement in all directions. The elastic modulus values for the Ag NW and Si substrate were set to the built-in values in Comsol, accounting for their temperature-dependent nature. More technical details can be found in Supporting Information File 1.

Molecular dynamics simulations were performed with the large-scale atomic/molecular massively parallel simulator (LAMMPS) [40]. Interactions between the atoms were governed by the embedded atom method (EAM) potential [41] for silver atoms. Visualization was performed with the Open Visualization Tool (OVITO) [42]. The system time step was 10 fs. More technical details can be found in Supporting Information File 1.

## Results and Discussion

### Heat treatment

#### First heating scheme

**Figure 3:**
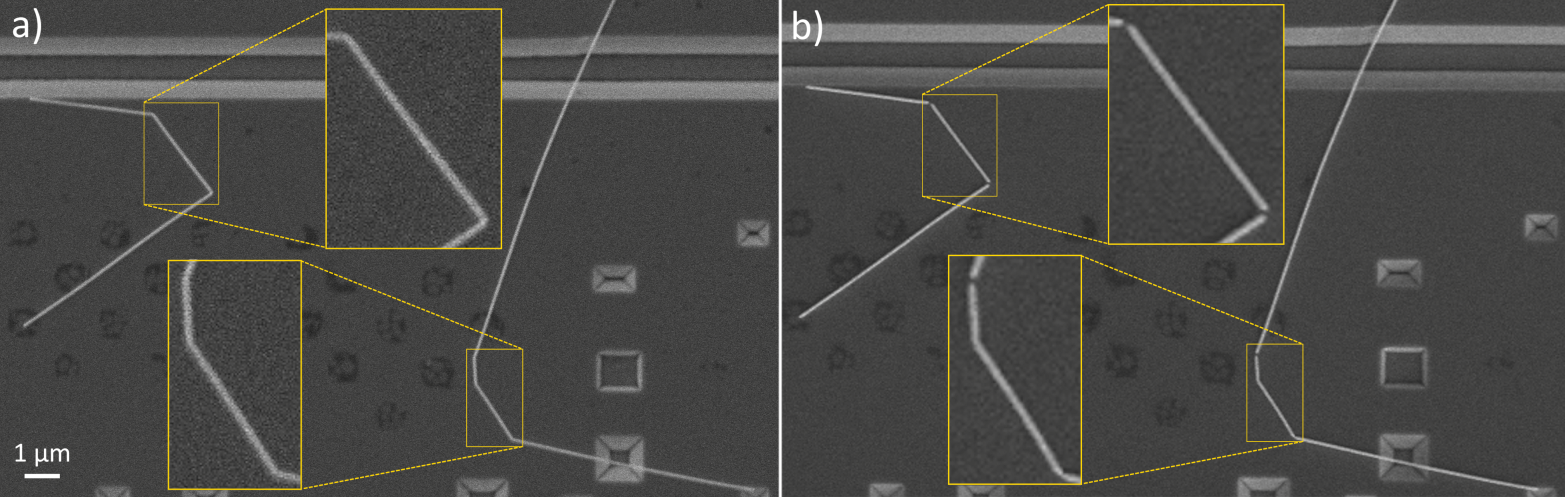
a) Ag NWs deformed after deposition onto a Si substrate. b) Splitting of the Ag NWs at the bending areas after four heating cycles (100, 150, 200, and 225 °C) in scheme 1. Note that darker structures on the substrate are from local modification of Si by the etchant and are flat. These structures are not important for the splitting of NWs. The location was chosen due to the ability to show several splits in one image.

No significant changes in the morphology of Ag NWs were detected for heat-treatment temperatures up to 275 °C. Starting from 300 °C, the first clear signs of diffusion in NWs appeared in the form of splitting at the places where NWs were partly broken during deposition (Figure 3). In addition, fusion at the intersections of two or more NWs (Supporting Information File 1, Figure S1) was observed, in agreement with other studies [27–28,30,43]. No difference in behavior between adhered and suspended parts of NWs regarding structural integrity was detected by this point. The only noticeable factor influencing the process was the diameter of NWs: in general, thinner NWs started to diffuse earlier.

**Figure 4:**
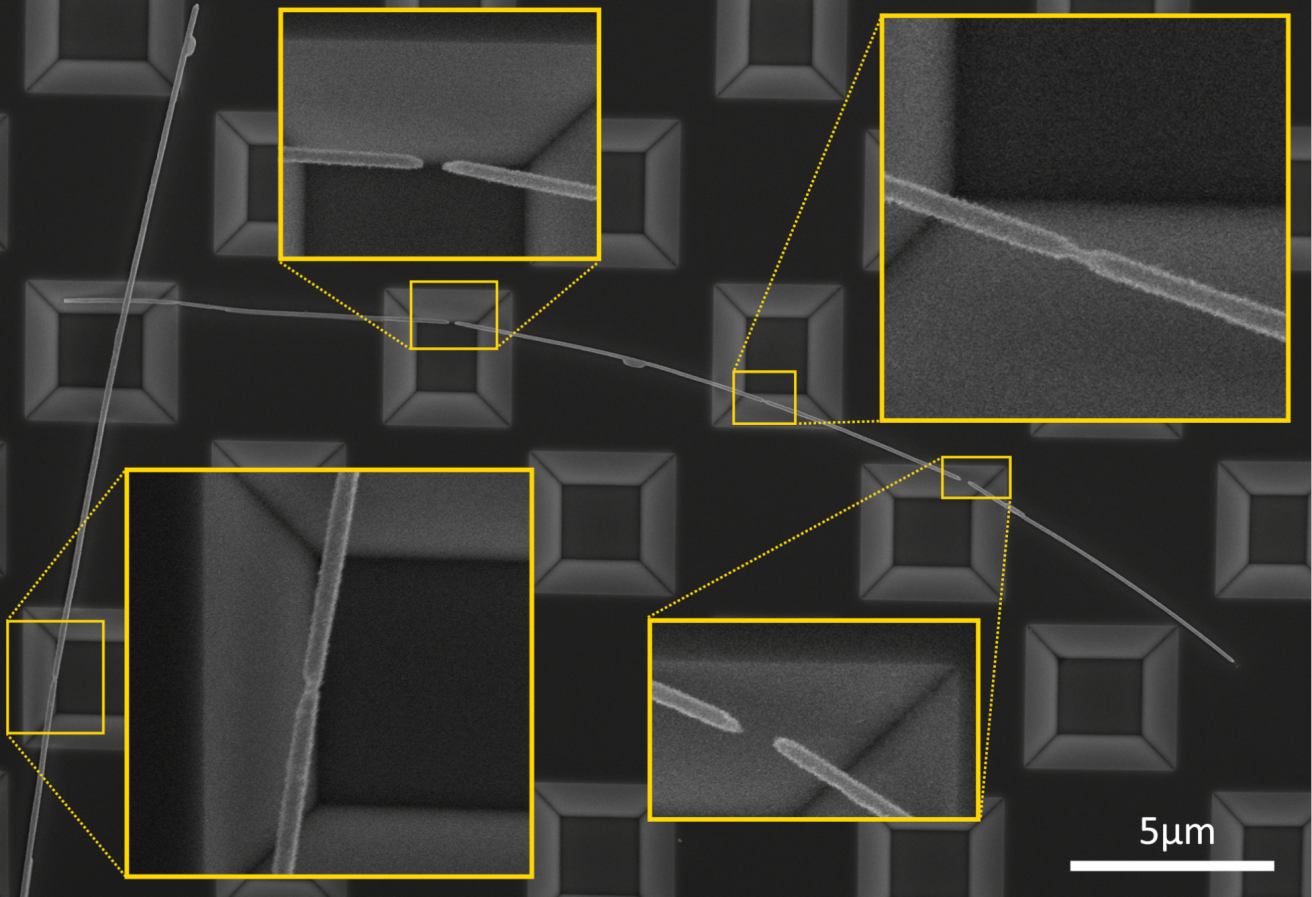
**:** Necking and splitting of Ag NWs in the heating scheme 1 after treatment at 375 °C.

In the temperature range from 350 to 375 °C, necking and complete splitting of Ag NWs approximately in the middle of suspended parts (Figure 4) was observed in many NWs. Starting from 400 °C, fragmentation of NWs spread to the adhered parts (Supporting Information File 1, Figure S2). From this finding, it can be concluded that in the heating scheme 1, adhered parts are more heat resistant and can withstand approximately 50 °C higher temperatures before fragmentation compared to the suspended parts. At 450 °C, most NWs were split in the suspended parts, and extensive fragmentation was also present in the adhered parts. Again, in general, thinner NWs were more prone to necking and splitting, while thicker NWs withstood higher temperatures.

#### Second heating scheme

**Figure 5:**
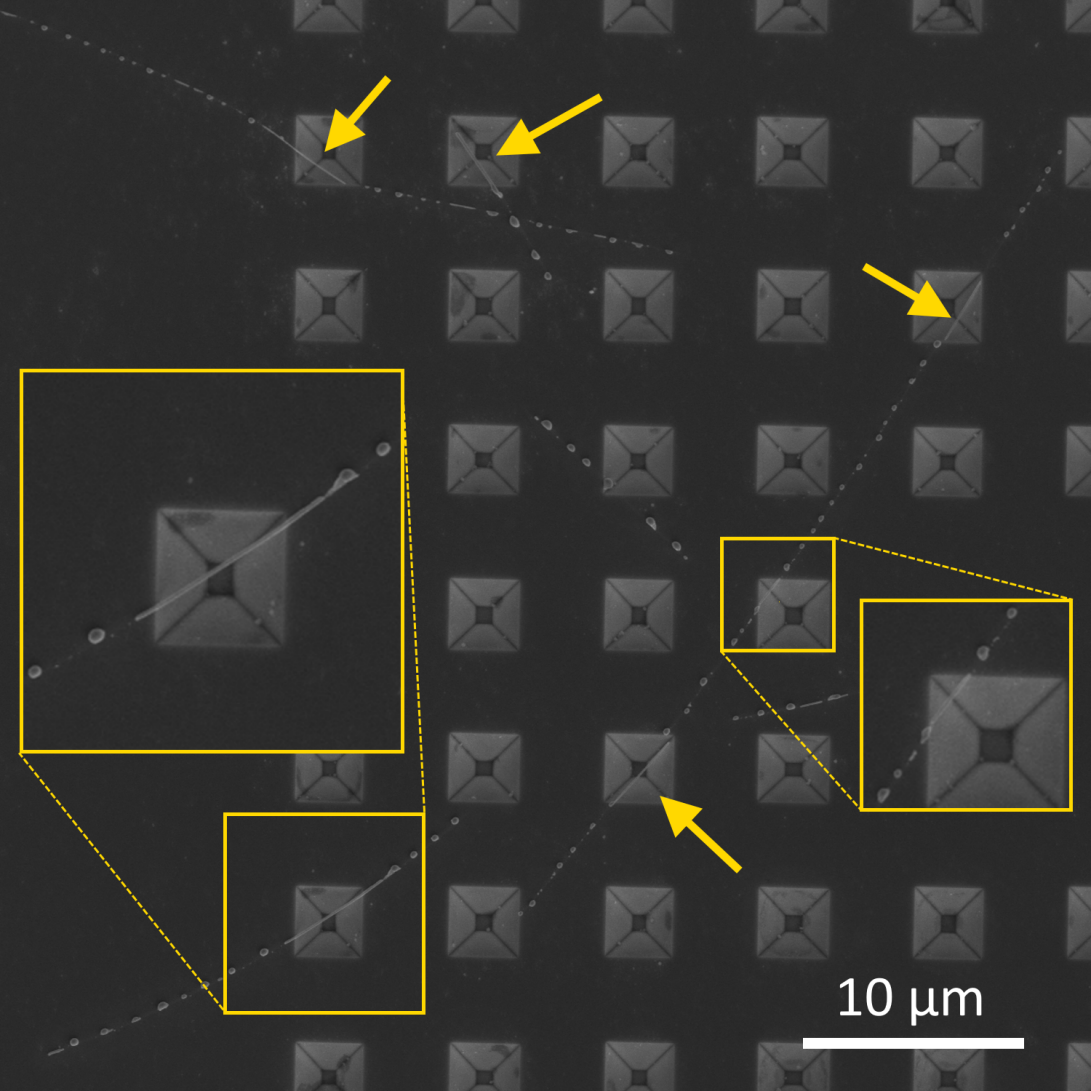
SEM images of Ag NWs after a single-step heat treatment (heating scheme 2) at 400 °C. Fragmentation of NWs happened almost exclusively in the adhered parts.

For scheme 2, it was possible to “catch” the early stage of fragmentation of NWs, which is essential for comparison of the behavior of adhered and suspended parts under heat treatment, in the temperature range of 375–400 °C. It was found that the behavior of partially suspended NWs in the heating scheme 2 was completely opposite to what we found in the heating scheme 1. Namely, extensive fragmentation occurred in the adhered parts while the suspended parts remained in one piece (Figure 5). The effect was most pronounced for the samples treated at 400 °C, therefore this temperature was chosen for main tests. Above that temperature, extensive fragmentation was observed in both adhered and suspended parts.

The heat-treatment experiments (both heating schemes) were repeated two times, each time on freshly made samples and following the same protocol of the heating scheme. Each time the results consistently demonstrated the tendency to split first in the middle of the suspended part for the heating scheme 1, and in the adhered part for the heating scheme 2.

**Figure 6:**
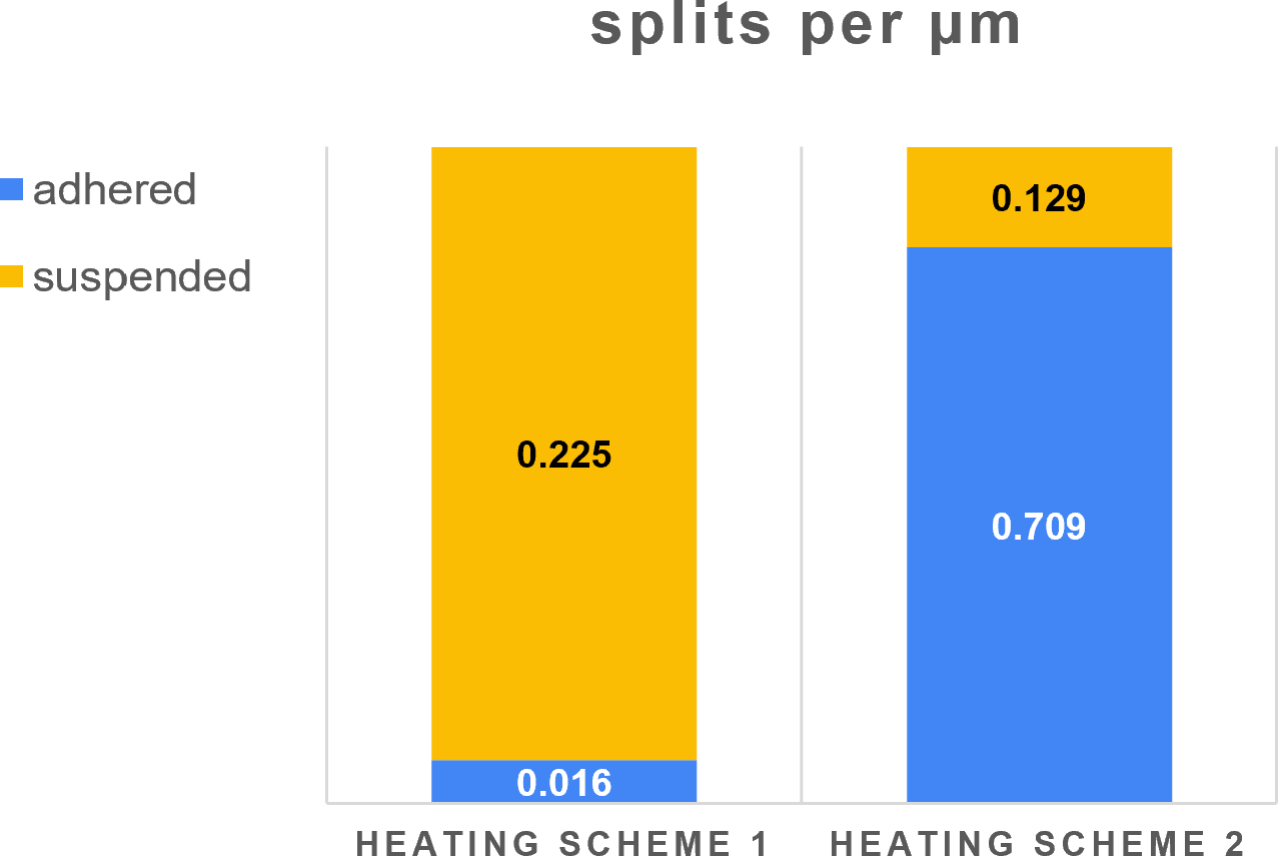
Average values of splits per length unit in adhered and suspended parts for both heating schemes.

To quantitatively describe the extent of splitting in the adhered and suspended parts of Ag NWs, we introduced two parameters: “splits per part”, which denotes the total number of split events separately for the adhered and suspended parts of each NW, and “splits per unit length”, which indicates the number of split events per length of either adhered or suspended part. The number of splits was calculated from SEM images of the large areas (approx. 120 × 80 µm) taken with maximum picture resolution (6144 × 4415). This ensured that there was no bias in choosing individual NWs for analysis, but all NWs in large areas are analyzed. Then, the average values for all analyzed NWs were separately calculated for each heating scheme. In total, 111 adhered and 101 suspended parts were analyzed for the heating scheme 1, and 87 adhered and 64 suspended parts for the heating scheme 2. Normalized results of the statistical analysis are given in Figure 6. For the heating scheme 1, the extent of fragmentation in the suspended parts is one order of magnitude higher than that for the adhered parts, while a totally opposite behavior is observed for the heating scheme 2.

### Inner structure of Ag nanowires

**Figure 7:**
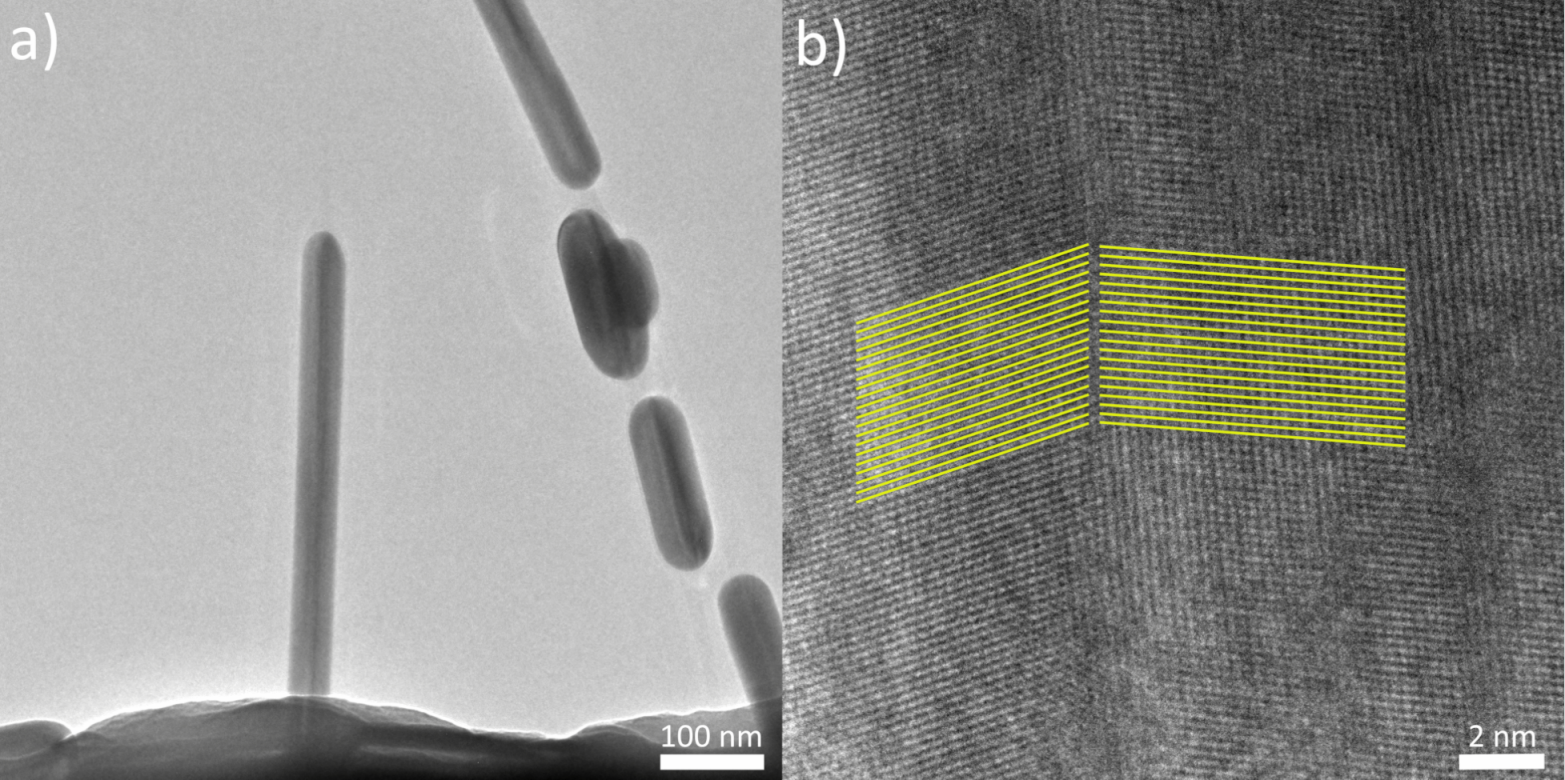
a) TEM images of Ag NWs after single-step (scheme 2) heat-treatment. b) Inset to one of the segments. Dark lines in the middle of NWs correspond to a twin border between two crystal segments.

Silver NWs used in the present study have a five-fold twinned crystal structure resulting in a pentagonal cross-section. Since pentagonal symmetry is a “forbidden” symmetry in crystallography, five-fold twinned crystals unavoidably have inner strains [44]. This could potentially be one of the driving forces leading to heat-induced fragmentation of Ag NWs as the mechanism of stress release, and could potentially involve recrystallization into single crystals. To test this hypothesis, we repeated experiments on TEM grids with subsequent observation of the crystal structure via TEM. It was found that the pentagonal structure is preserved even for small fragments of NWs that were split as a result of heat treatment as shown in Figure 7 (note that the fragments are kept in place due to contact with the thin carbon membrane of the TEM grid). These findings suggests that heat-induced morphological changes in Ag NWs occur via surface diffusion without the loss of crystal structure.

### Hypothesis and simulations

#### Hypothesis and finite element method simulations

According to our understanding, there are several key aspects that should be considered in order to explain the results of the heat-treatment experiments. Firstly, Ag has almost an order of magnitude higher thermal expansion coefficient compared to that of Si (18.9 vs 2.8 × 10^−6^ m/(m·°C)) [45]. Secondly, from nanomanipulation experiments previously performed on similar Ag NWs [8], we know that the strength of the contact between Ag NWs and Si substrate can exceed the ultimate strength of Ag NWs.

Based on these facts, we expect that the thermal expansion of Ag NWs will compete with friction forces between the NWs and Si substrate, causing significant mechanical stresses inside the NWs, especially at the interface between the two materials. This may serve as a driving force for the redistribution of Ag atoms and splitting of the adhered part of a NW into shorter fragments as a mechanism for stress mitigation. For the suspended parts, the thermal expansion should result in deformation. To estimate the extent and distribution of the heat-induced mechanical stresses in a partially suspended NW, we performed corresponding FEM simulations (Supporting Information File 1, Figure S3). According to simulations, the highest stresses (up to 1.5 × 10^9^ N/m^2^) are concentrated at the interface between the adhered part and the Si substrate, followed by the stresses in the middle of the suspended part (up to 1 × 10^9^ N/m^2^).

In this simplified idealistic model, the contact between a NW and Si is absolutely rigid, preventing any slippage or plastic deformation at the interface. Moreover, FEM simulations do not include possible rearrangement of atoms that can occur in the real system as a mechanism of stress mitigation. The model is completely elastic, and the simulated processes are fully reversible. Therefore, the results of FEM simulations should only be treated as a simplified qualitative illustration of the thermomechanical processes that may occur in the real system. Nevertheless, such results may give an additional hint for understanding the mechanisms responsible for splitting in different configurations.

**Figure 8:**
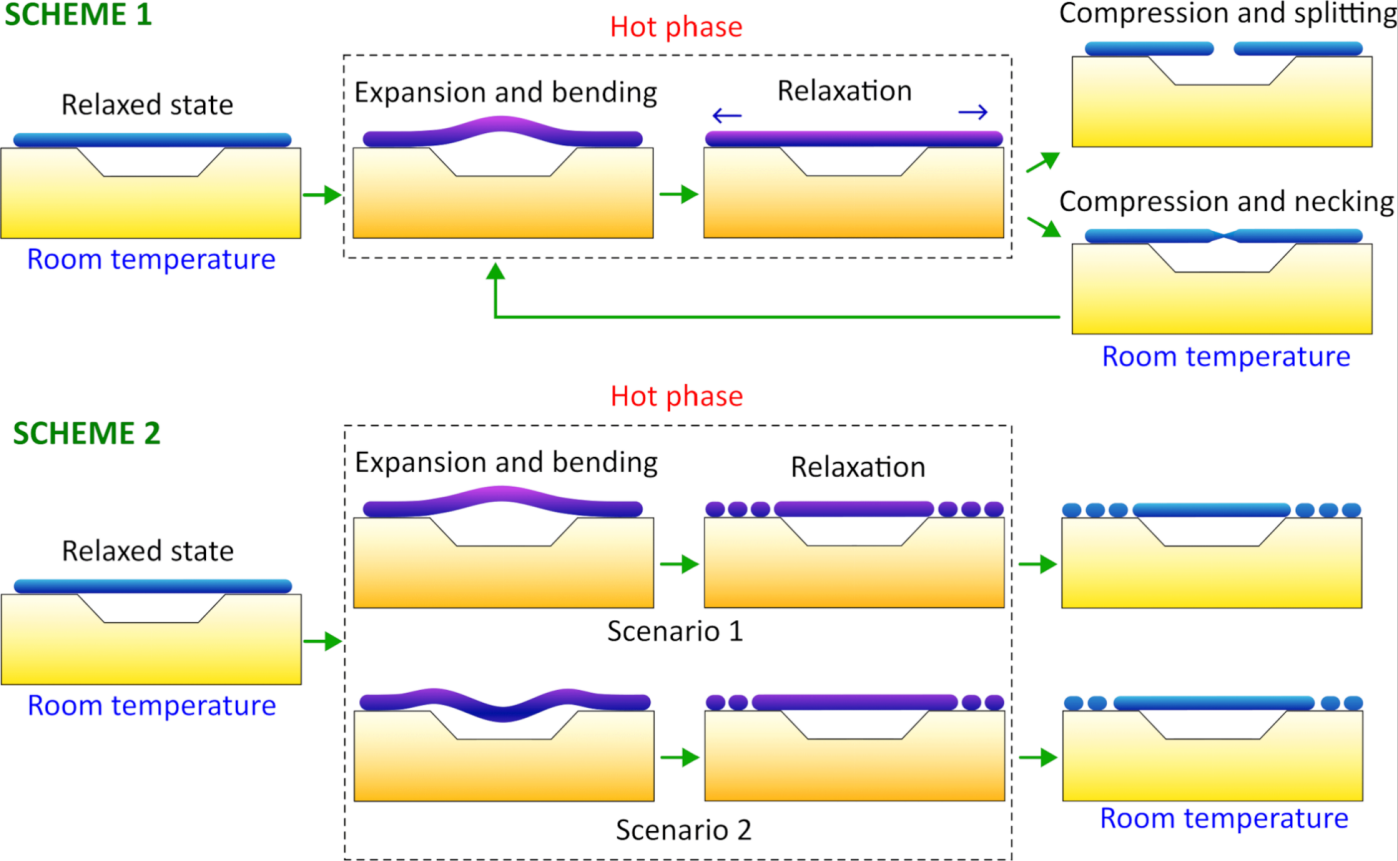
Schematic representation of the deformation and fragmentation of NWs during the heat treatment.

In particular, we can speculate that repeated heat-induced bending of the suspended part in scheme 1 can lead to fatigue and formation of defects in the middle of the suspended part. This effect should be even more pronounced if we assume that a NW is deformed and stressed only in the early stage of the hot phase followed by a gradual relaxation of the NW by the means of heat-enhanced rearrangement of Ag atoms at the contact with the substrate as a mechanism of stress mitigation. In other words, the NW will “slide” along the substrate to adapt to heat-induced elongation without deformation. When the sample is removed from the hot furnace, rapid cooling and thermal contraction should give rise to tensile stresses inside the NW, especially considering the greatly reduced mobility of Ag atoms at lower temperatures preventing slippage of the NW relative to the Si substrate. As a result, defects and necking develop in the middle of the suspended part, which agrees with the experimentally observed necking in scheme 1 (Figure 4). As shown above (Figure 3), heat-induced splitting tends to first occur at regions that have significant structural defects, which is also discussed in more detail by Mayoral et al. [46] and Sun et al. [47]. According to the latter, atoms in a NW tend to diffuse away from the regions of critical defects. Diffusion is expected to start mainly at the surface resulting in the thinning of the NW in the middle of the suspended part. It means that a further heat treatment will result in the splitting of the necking region before the NW splits anywhere else. Therefore, we have a potential mechanism responsible for splitting of NWs in the suspended part in scheme 1. The process is schematically shown in Figure 8 (scheme 1).

For scheme 2 the situation is simpler. Since there are no heating cycles involved, the suspended parts do not experience repeated compression and tensile stresses. The adhered parts split into shorter fragments in a similar way as it generally happens with heated Ag and Au NWs on a flat Si substrate [27]. This process is commonly explained by the phenomenon known as Rayleigh instability [48–50]. According to our understanding, stresses that arise at the interface between the NW and the substrate due to the difference in thermal expansion, also play a crucial role in the process. Otherwise, the fragmentation would be similar regardless of the contact with the substrate. Therefore, in the experiments the suspended parts survive longer as they avoid interfacial stresses. Thus, fragmentation of NWs should be rather attributed to the interplay between heat-induced mechanical stresses and Rayleigh instability.

Note that, in some cases, the length of the survived part is longer than the length of the visible suspended part (i.e., it slightly spreads outside the hole). A possible reason is that the heat-induced bending can cause the detachment of the NW close to the hole similarly to peeling or crack formation and propagation. The process is schematically depicted as Scenario 2 in Figure 8 (scheme 2). In principle, Scenario 2 may also be realized for the scheme 1. However, it should not affect the outcome of the experiment since in any case defects caused by heating/cooling (hence, tensile compression) cycles will result in reduced thermal stability of the suspended part compared to the adhered part.

Note that, as mentioned earlier, the morphology evolution in the adhered parts is similar for both schemes. Namely, the fragmentation of adhered parts into shorter particles at higher temperatures will be observed in both cases. The main differences are observed in suspended parts at transitional temperatures when the first morphological changes in individual NWs are clearly visible.

#### Molecular dynamics simulations

**Figure 9:**
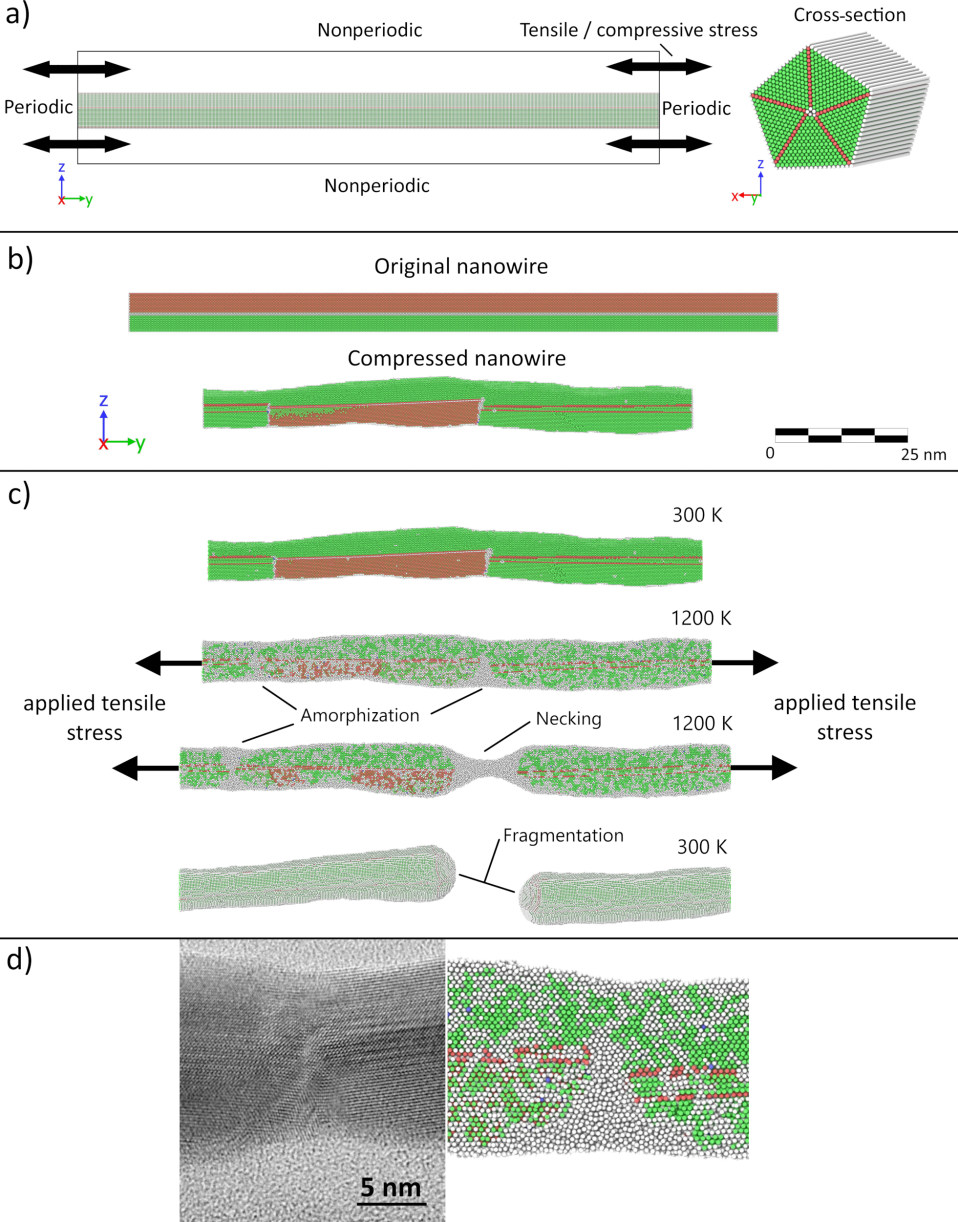
Molecular dynamics model of the NW: (a) initial conditions, (b) results of the compression cycle, (c) results of the tensile deformation; (d) amorphization of the central part of the NW as a result of heat-treatment cycles: TEM image (left) and MD simulation frame taken when the structure had turned amorphous but not yet broken (right). Color code: green – FCC lattice, red – HCP, blue – BCC, white – amorphous.

To further investigate the role of stress-induced defects in the splitting of NWs at elevated temperatures in scheme 1, we performed MD simulations (see detailed description in Supporting Information File 1). A NW was simulated as a periodic prismatic rod with a pentagonal cross-section and a five-fold twinned inner structure (Figure 9). A series of heating and cooling cycles was applied, accompanied by compression and tensile deformations along the NW. The heating cycle induced the formation of defects, and amorphous regions were the most pronounced in the central part of the NW with subsequent evolution into necking and finally splitting (Figure 9b, Figure 9c).

It should be noted that the goal of the MD simulations was only to qualitatively evaluate the role of the thermomechanically induced defects in the fragmentation of five-fold twinned Ag NWs. Therefore, the model has significant differences and simplifications compared to the real experiment. Namely, an isolated NW was simulated, which excludes the role of the substrate in the process. The diameter of the NW model was an order of magnitude smaller compared to that of the experiment. To compensate for the drastic difference in the timescales between the experiment (minutes) and simulations (nanoseconds), significantly higher (but still below the melting point) temperatures were used in the simulations. Nevertheless, MD results qualitatively agree well with the TEM images of the NWs which underwent several heating/cooling cycles (Figure 9d).

### Other heat-induced effects

In addition to the aforementioned effects, it was found that in both schemes the heat treatment sometimes caused residual elongation and bending of the suspended parts. One example is given in Supporting Information File 1, Figure S4 showing a significant bending in the suspended part in the heating scheme 2. The onset temperature of this phenomenon is difficult to determine as the deformation can be below the detection limits of SEM. Moreover, in SEM we only see the 2D projection normal to the electron beam. If NWs are bent out of the substrate plane (upwards or downwards) it will not be visible in the SEM image.

If a NW is contacting one or more NWs, then heat-induced redistribution of silver atoms between the NWs is often observed, resulting in thickening and shortening of all the contacting NWs (Supporting Information File 1, Figure S5). Some NWs may completely disappear in this process, being absorbed by the neighbors, as also described by Mayoral et al. [46]. In this work we focused on individual NWs; therefore, detailed analysis of the NW bundles lies outside the scope of the present study.

## Conclusion

In this work, we investigated the effect of heat treatment on pentagonal Ag NWs that are only partially in contact with the substrate. We deposited Ag NWs on patterned Si substrates which had arrays of holes of different sizes so that some NWs were partially suspended over the holes. We found that if the temperature is increased in a stepwise manner (in 25 °C increments) cycles of rapid heating followed by rapid cooling to room temperature after each cycle (scheme 1), then at 375 °C, nanowires start to split in the middle of the suspended parts, while the adhered parts remain mostly intact (0.225 vs 0.016 splits per µm). If the temperature is directly increased from room temperature to 375 °C or 400 °C (scheme 2), the opposite behavior is observed. Namely, most suspended parts remain intact, while extensive fragmentation occurs in the adhered parts (0.129 vs 0.709 splits per µm). At temperatures above 400 °C both adhered and suspended parts are involved in fragmentation. According to our understanding, such behavior is related to mechanical stresses that arise in nanowires as an interplay between thermal expansion and frictional forces. The finite element method and molecular dynamic simulations gave good qualitative agreement with the experimental observations. Our finding suggests that heat-induced fragmentation of metallic nanowires in general cannot be purely explained by the commonly accepted Rayleigh instability model, but should include effects related to the interaction with the substrate.

## Supporting Information

File contains additional SEM and TEM images, as well as additional details of FEM and MD simulations.

File 1 Additional images and simulations.

## Data Availability

The data that supports the findings of this study is available from the corresponding author upon reasonable request.
