# Supplementary material for: Heat-induced morphological changes in silver nanowires deposited on a patterned silicon substrate
Source: Beilstein J Nanotechnol. 2024 Apr 22;15:435–46. doi: 10.3762/bjnano.15.39 (PMC11070972; doi:10.3762/bjnano.15.39)
Supplement: File 1 — Additional images and simulations. [file Beilstein_J_Nanotechnol-15-435-s001.pdf]

BEILSTEIN JOURNAL OF NANOTECHNOLOGY

Supporting Information

for

Heat-induced morphological changes in silver nanowires deposited on a patterned silicon substrate

Elyad Damerchi, Sven Oras, Edgars Butanovs, Allar Liivlaid, Mikk Antsov, Boris Polyakov, Annamarija Trausa, Veronika Zadin, Andreas Kyritsakis, Loïc Vidal, Karine Mougin, Siim Pikker and Sergei Vlassov

Beilstein J. Nanotechnol. **2024,** *15,* 435–446. doi:10.3762/bjnano.15.39

Additional images and simulations

License and Terms: This is a supporting information file under the terms of the Creative Commons Attribution License (https://creativecommons.org/licenses/by/4.0). Please note that the reuse, redistribution and reproduction in particular requires that the author(s) and source are credited and that individual graphics may be subject to special legal provisions.
The license is subject to the *Beilstein Journal of Nanotechnology* terms and conditions: (https://www.beilstein-journals.org/bjnano/terms)

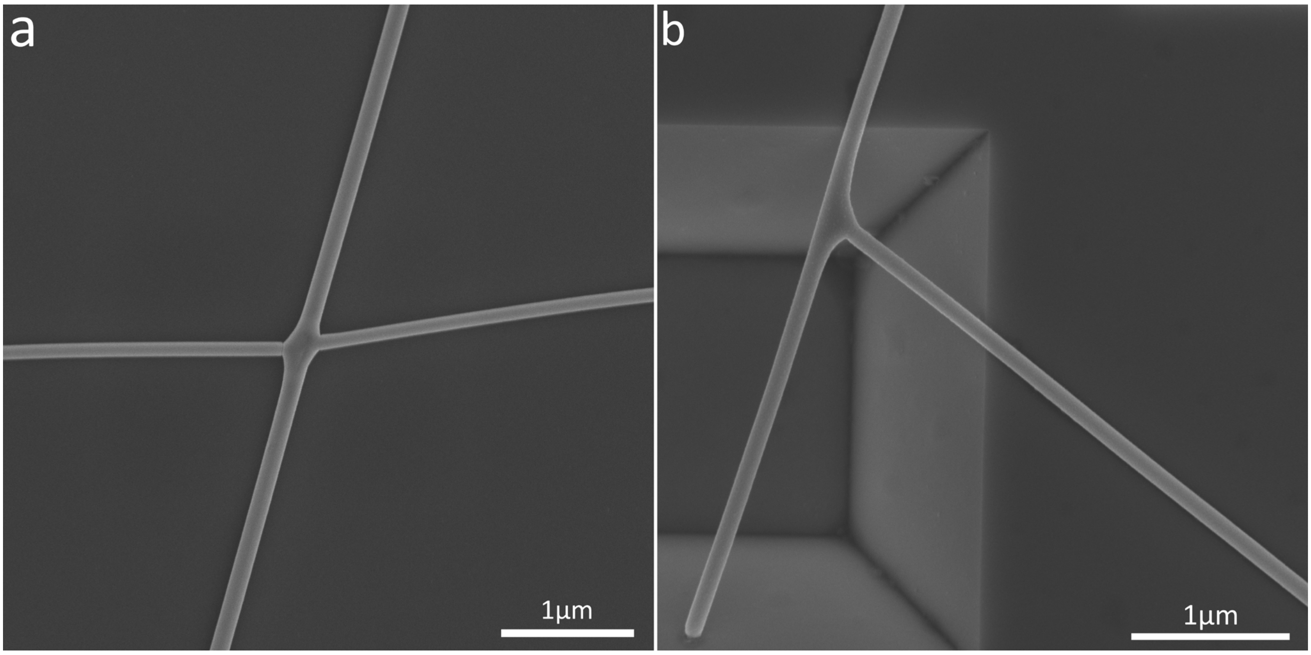

Figure S1: Intersections of nanowires after heat treatment at 300 C. (a) non-suspended, (b) suspended.

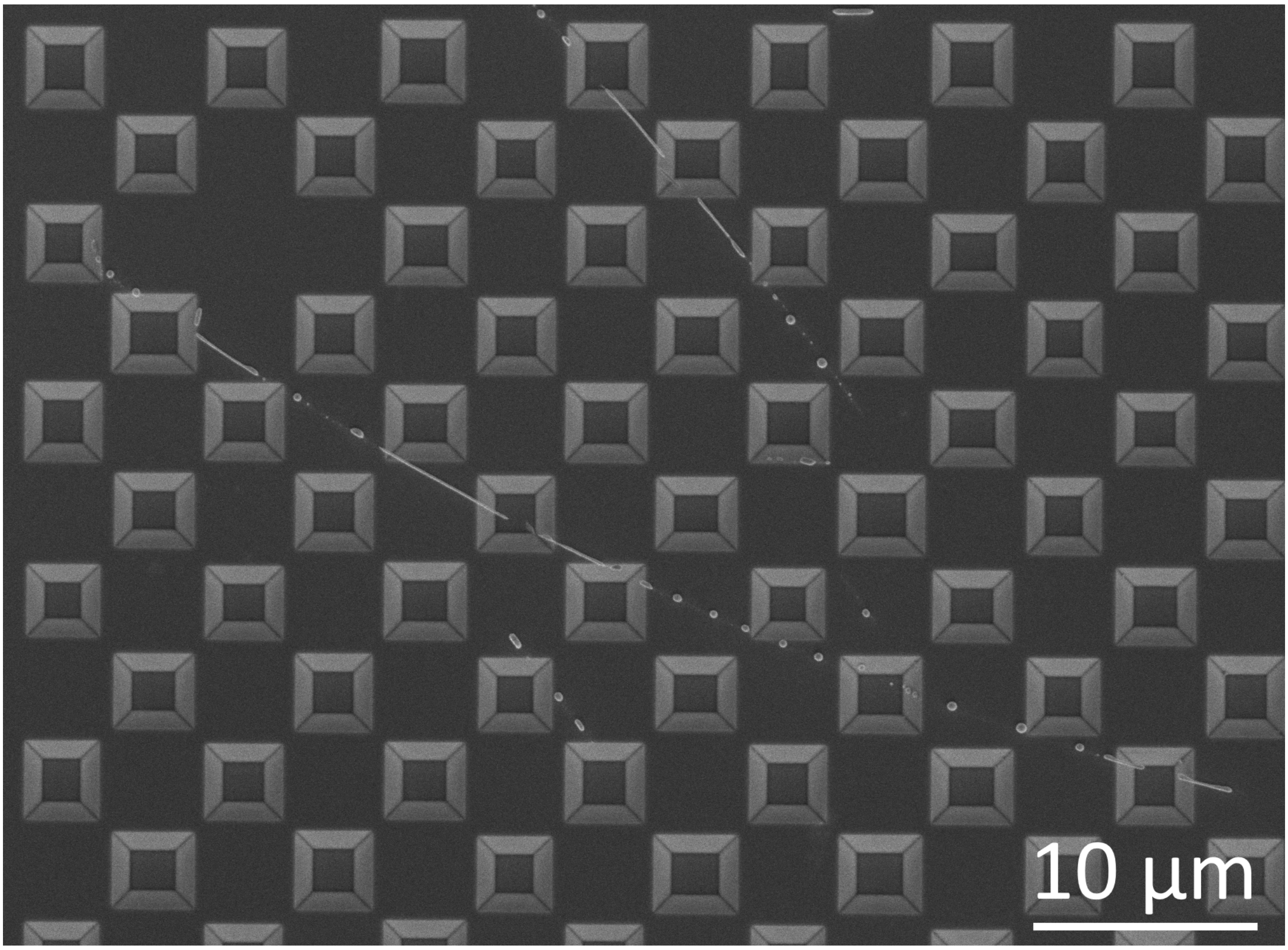

Figure S2: Scheme nr 1 - from 400 C fragmentation starts spreading to the adhered parts.

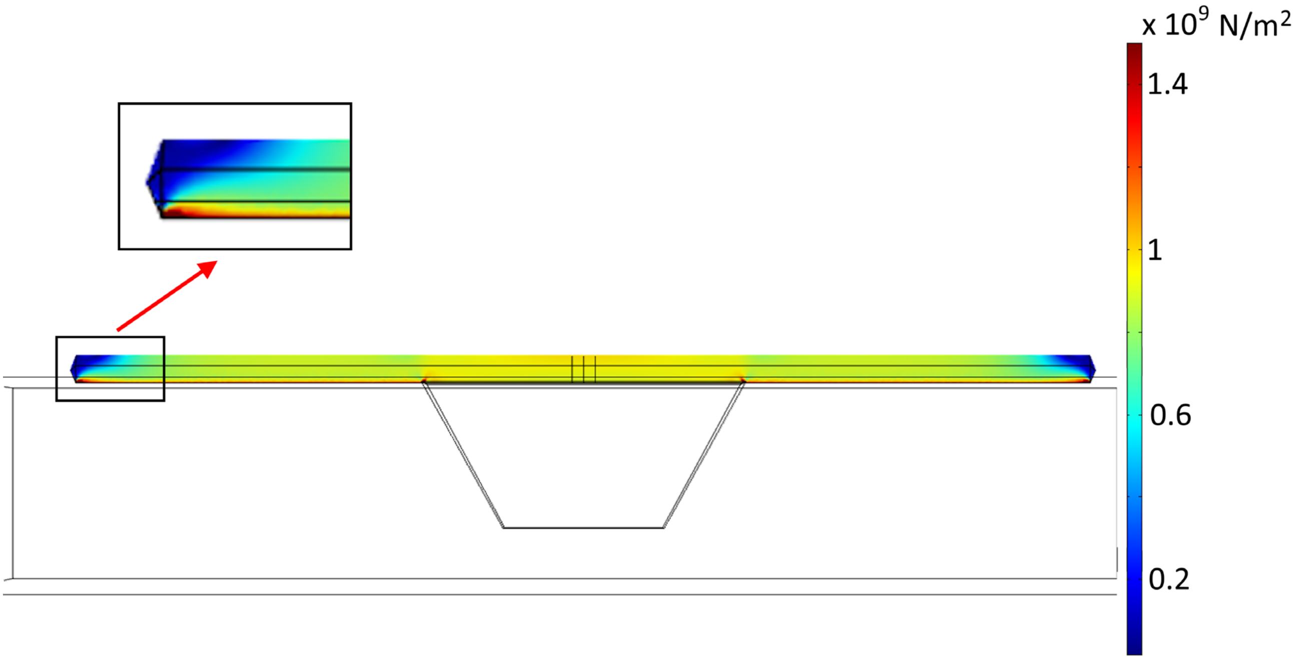

Figure S3: FEM simulation of mechanical stresses (von Mises) generated in partly suspended Ag NWs as a result of thermal expansion caused by heating from room temperature to 400 °C. Adhered parts are rigidly fixed to the Si substrate.

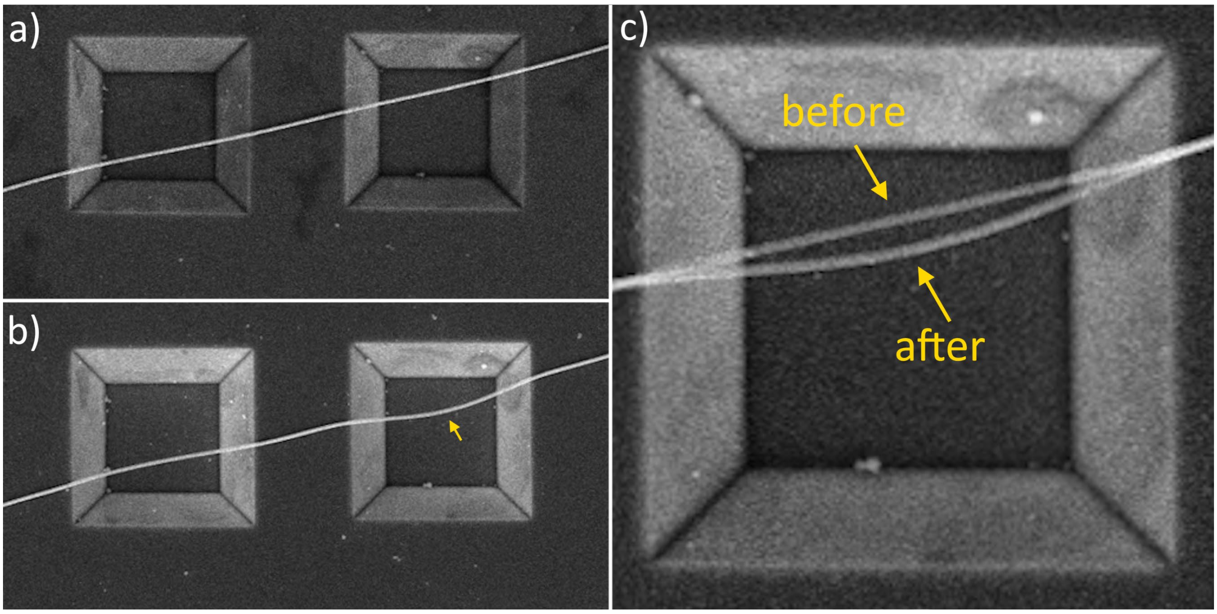

Figure S4: SEM image showing the bending of the Ag NW as a result of heat treatment in scheme 2. Image c) is made by superimposing a) and b) images.

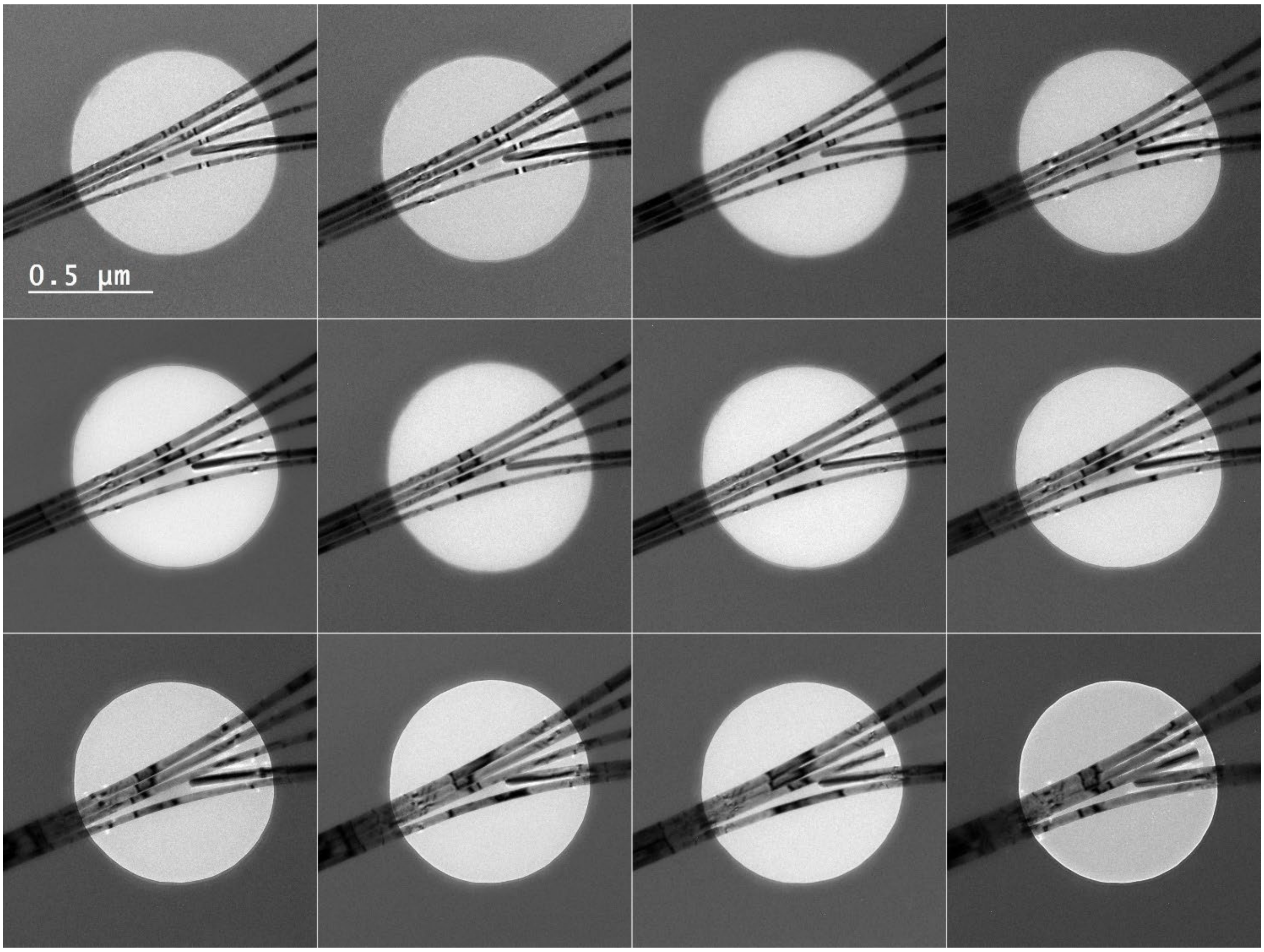

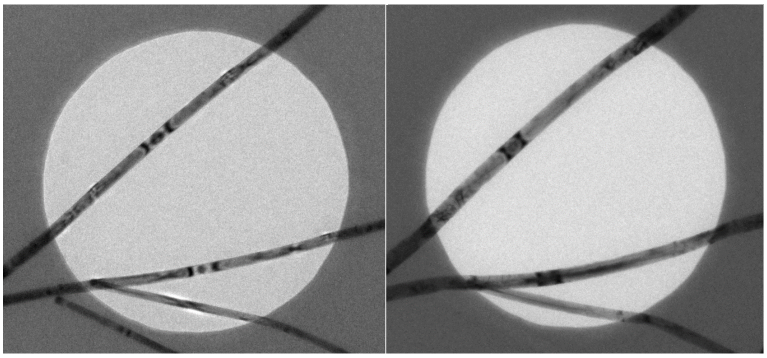

Figure S5: Redestribution of the material between contacting Ag NWs as a result of a heat treatment.

Finite element method simulations

The thermal expansion behavior of a Ag NW was accurately characterized through the utilization of Comsol Multiphysics 5.6. This comprehensive model encompassed a stationary study, leveraging both solid mechanics and heat transfer in solids for a thorough analysis. The structural configuration involved a pentagonal Ag NW positioned above a rectangular hole on an Si substrate. The NW was securely affixed to the substrate, while the

overhanging segment retained freedom of movement in all directions. To enhance geometric accuracy, the edges of the rectangular hole were seamlessly transformed into circular fillets with a radius of 20 nm.

The model delineated two distinct domains, namely the Ag NW and the Si substrate, each meshed with a different number of tetrahedral elements – approximately 96,000 for the Au NW and 31,000 for the Si substrate. The elastic modulus values for the Ag NW and Si substrate were set to the built-in values in Comsol, accounting for their temperature-dependent nature. The initial conditions of the simulation dictated a uniform temperature of 293.15 K across the entire geometry, with the simulated temperature reached 673.15 K.

Employing the stationary solver, the simulation successfully replicated the thermal expansion phenomenon at the final temperature. Visualization of the von Mieses stress distribution facilitated the identification of sections within the nanowire experiencing the highest stress concentration. This meticulous approach enhances our understanding of the intricate thermal expansion dynamics in the gold nanowire system.

Molecular dynamics simulations

The aim of the molecular dynamics simulation is to create a comparable geometry and conditions to the experiments in order to induce similar morphological changes in the nanowire. Using pre-constructed sections of the nanowire and applying compressive and tensile stress, we created similar necking and fragmentation of the wire to propose a possible mechanism for its occurrence.

Compressive stress simulation

Molecular dynamics simulations were performed with the Large-scale Atomic/Molecular Massively Parallel Simulator (LAMMPS) [1]. Interactions between the atoms were governed by the embedded atom method (EAM) potential [2] for silver atoms. Visualization was performed with the Open Visualization Tool (OVITO) [3]. The system time step was 10 fs.

Simulation geometry and conditions are illustrated in Figure 9a in the main text. A prefabricated pentagonal NW with a diameter of 7.6 nm was placed in a simulation cell with periodic boundaries in the y direction and nonperiodic in the x and z directions. The system length in the y direction was 140 nm. The system was at first relaxed for 50 ps at 10 K with NpT isothermal–isobaric ensemble. After that, the temperature and applied compressive pressure were gradually raised to 1185 K and 95 MPa during a period of 1500 ps. After reaching set values, the wire was relaxed for 100 ps at constant values, after which temperature was controlled for 4 ns by the NVT isothermal ensemble. This allowed the wire to reorganize its geometry in the, at first, highly compressive conditions without altering the box size. Afterwards, the pressure on the wire was reduced to 0 Pa N and then raised to 55 MPa during 500 ps, relaxed at a constant setting for 50 ps, and controlled by NVT ensemble for 3500 ps. This was cyclically repeated for 45, 40, 40, and 35 MPa. Afterwards, the wire was relaxed and cooled to 300 K.

This produced a shortened NW (Figure 9b in the main text) with internal defects that could be compared in tensile simulations to an ideal NW. The length of the wire after compression was 126 nm. Applied compressive stress simulated the conditions of the wire during the heating process when thermal expansion compresses it. The result is the creation of defects that contribute heavily to the fragmentation of the wire during the application of tensile stress during the cooling cycle.

Tensile stress simulations

The tensile simulations were performed with the same basic settings as the compressive simulations. Instead of the ideal NW, the result of the compressive test took its place. The system was relaxed at 300 K and then heated to 1200 K with an applied tensile pressure from 0 to 200 MPa with NpT isothermal–isobaric ensemble during 1000 ps. Temperature close to the melting point of the wire was chosen so that the possible diffusion speed and fragmentation were as fast as possible. Then, the pressure was slowly increased to 452 MPa during 2000 ps with constant temperature. After 1500 ps of NVT ensemble control, the wire was cooled and relaxed to 300 K. Previous maximum pressures were found by experimenting with simulations to visualize a breakup but not over or understretch the wire. Due to the short simulation period reasonably available, the task was to completely break the internal structure of the NW at one point and then see if diffusion results in necking or repairing of the wire. The result was, at first, the amorphization of the Ag structure at one of the defect points, and the subsequent diffusion-induced necking and breakup of the wire.

This modeled the tensile stress induced in the wire during the cooling cycle of the experiment due to thermal contraction. Due to the previously created defect points in the wire, the tensile stress is able to contribute to the amorphization of the structure after which the diffusion of Ag atoms results in the fragmentation of the wire. Combined with the previous process of compression, this cycle is a possible mechanism for the morphological changes seen in experiments on the substrate.

A similar scheme without applied pressure was also used for the previous pre-fabricated ideal NW to gain comparative results. The same heating regime produced no necking or fragmentation of the NW, which points to the importance of pre-existing defects in the breakup of NWs. Using LAMMPS code to calculate and save to an external file the atomic potential energy of the two systems, the difference between the two systems can be visualized. For some time, the atomic energy of the tensile wire remains constant, but at one moment, the energy starts increasing, alluding to a structural change in the wire (Figure S6). This indicated the ongoing diffusion and eventual fragmentation of the wire. The energy level of the regular wire, however, remains constant. The small difference in the comparative stable values at the beginning can be explained by the different thicknesses and already defective structure of the wire.

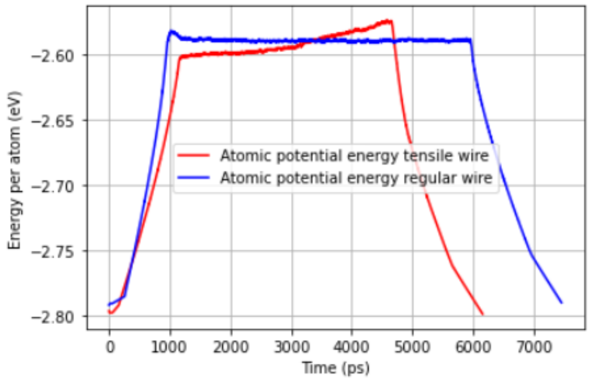

Figure S6: Comparison of the atomic potential energy graphs for defect wire under stress and unstressed wire

Crystal structure after cooling

To better visualize the structure of the NW and provide a comparison with TEM pictures, a snapshot of the wire was taken at a certain time when the structure had turned amorphous but not yet broken. After that, the wire was cooled to 300 K.

Cooling was repeated with differently initialized speeds to account for the stochastic nature of the process. Every wire exhibited at least one area with crystal structure formation equivalent to the structures seen in TEM images of Ag NW after several heat-treatment cycles (Figure S7 here and Figure 9d in the main text).

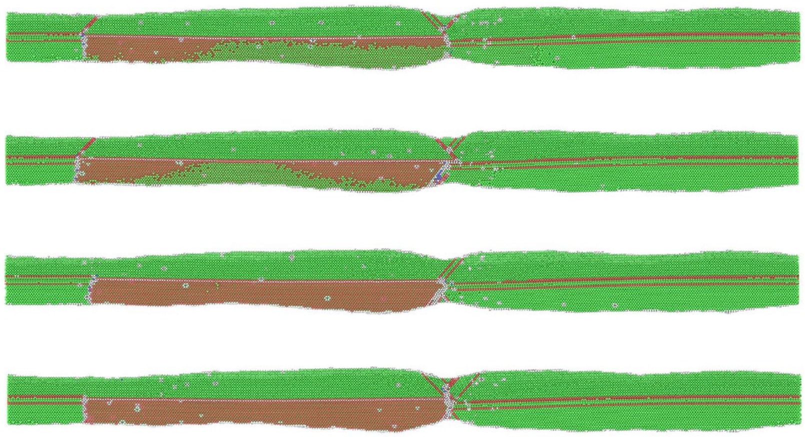

Figure S7: Comparison of cooled NWs at 300 K with different initialization seeds producing triangular defects.

References

[1] Plimpton S 1995 Fast parallel algorithms for short-range molecular dynamics J. Comput. Phys. 117 1–19.

[2] P.L. Williams, Y. Mishin, and J.C. Hamilton (2006), "An embedded-atom potential for the Cu-Ag system", *Modelling and Simulation in Materials Science and Engineering*, **14(5)**, 817-833. DOI:10.1088/0965-0393/14/5/002

[3] Stukowski A 2010 Visualization and analysis of atomistic simulation data with OVITO—the open visualization tool Modelling Simul. Mater. Sci. Eng. 18 015012.
